# UCN-01 induces S and G2/M cell cycle arrest through the p53/p21^waf1^ or CHK2/CDC25C pathways and can suppress invasion in human hepatoma cell lines

**DOI:** 10.1186/1471-2407-13-167

**Published:** 2013-03-28

**Authors:** Guoyi Wu, Linan Xu, Nan Lin, Bo Liu

**Affiliations:** 1 Department of General Surgery, the Lingnan Hospital, the Third Affiliated Hospital, Sun Yat-Sen University, GuangZhou, 510630, PR China; 2 Department of Gynecology and Obstetrics, the First Affiliated Hospital, Sun Yat-Sen University, GuangZhou, 510089, PR China; 3 Department of Hepatobiliary Surgery, the Third Affiliated Hospital, Sun Yat-Sen University, GuangZhou, 510630, PR China

**Keywords:** 7-hydroxystaurosporine (UCN-01), G2/M arrest, S arrest, Hepatocellular carcinoma, Anti-cancer drug, Beta-catenin, Tumour invasion and migration

## Abstract

**Background:**

UCN-01 (7-hydroxystaurosporine), a protein kinase inhibitor, has attracted a great deal of attention as a potent antitumour agent. Several clinical trials of UCN-01 alone or in combination with other agents for different tumour types are currently underway, and some of these trials have had positive results. Hepatocellular carcinoma has high incidence rates and is associated with poor prognosis and high mortality rates.

**Methods:**

Three different hepatoma cell lines (Huh7, HepG2, and Hep3B) were treated with different concentrations of UCN-01, and the anti-tumour effects of UCN-01 were evaluated. Following UCN-01 treatment, cell growth was measured using an MTT assay, cell cycle arrest was assayed using flow cytometry, and the mechanisms of cell cycle arrest and invasion inhibition were investigated through western blotting and a Matrigel invasion assay.

**Results:**

After a 72-h UCN-01 treatment, the growth of different hepatoma cell lines was significantly inhibited in a dose-dependent manner, with IC50 values ranging from 69.76 to 222.74 nM. Flow cytometry results suggested that UCN-01 inhibits proliferation in the hepatoma cells by inducing S and G2/M phase arrest, but not G1/S arrest, which differs from previous reports that used other tumour cell lines. Western blot results illustrated that UCN-01 induces a G2/M phase arrest, regardless of the status of the p53/P21^waf1^ pathway, whereas the CHK2/CDC25C pathway and the p53/p21^waf1^ pathway were involved in the UCN-01-induced S phase arrest. UCN-01 remarkably inhibited Huh7 cell invasion in a time-dependent manner. Suppression of Huh7 cell invasion may be due to the down-regulation of phosphorylated β-catenin by UCN-01.

**Conclusions:**

These findings suggest that UCN-01 induces hepatoma cell growth inhibition by regulating the p53/p21^waf1^ and CHK2/CDC25 pathways. Suppression of Huh7 cell invasion by UCN-01 may be due to the down-regulation of phosphorylated β-catenin. These data lend support for further studies on UCN-01 as a promising anti-HCC candidate.

## Background

Although substantial efforts have been made to improve the overall therapeutic outcomes for liver cancer, hepatocellular carcinoma (HCC) remains the fifth most common and the third most deadly cancer in the world [1]. If HCC is detected early, surgical resection and liver transplantation are curative options. However, most patients have no symptoms until the disease is in an advanced stage, precluding surgical treatment [2,3]. Many conventional anticancer treatments kill cells irrespective of whether they are normal or cancerous; therefore, patients suffer adverse side effects due to healthy cell loss. For this reason, new anticancer drugs are required.

UCN-01 (7-hydroxystaurosporine), both alone and in combination with chemotherapeutic agents and ionising radiation, is currently being evaluated in clinical trials as an antineoplastic agent [4]. UCN-01 has antiproliferative activity and is well tolerated both *in vitro* and *in vivo*. This antiproliferative activity may be through protein kinase C inhibition [5]. UCN-01 can enhance the cytotoxicity of chemotherapeutic agents through several potential mechanisms including inhibition of Chk1 [6]. In addition, UCN-01 can abrogate cell cycle arrest independent of p53 and p21^waf1^[7-9].

Regulation of cell growth is mainly controlled through cell cycle control mechanisms. Many cytotoxic compounds and DNA damaging agents induce cell cycle arrest [10,11]. In fact, many anti-cancer agents act by inducing cell cycle arrest. Progression of eukaryotic cells through the cell cycle is orchestrated by the sequential activation and inactivation of cyclin-dependent kinases (CDKs), which are associated with their respective cyclin subunits [12,13]. In addition, cell cycle progression is regulated by the relative cellular concentrations of CDK inhibitors. The Cip/Kip family members include proteins, such as p21^WAF1^, that bind to cyclin/CDK complexes and prevent kinase activation, subsequently blocking cell cycle progression at G1 [14,15]. In turn, activated Chk2 phosphorylates and inactivates the Cdc25c phosphatase, maintaining CDC2 in its phosphorylated-inactive form and leading to G2/M arrest [16,17]. Furthermore, Cdc25c expression is strongly associated with metastatic disease in HCC patients. Cdc25c inhibitors inhibit HCC cell growth by specifically delaying progression through S phase, most likely because cyclin A is not induced, which results in decreased Cdk2 and Cdc2 kinase activities [18]. The cyclin A was detected but the result was negative.

Although a number of studies have attempted to elucidate the mechanism of UCN-01 function, there have been no reports on the effects of UCN-01 on hepatoma cells. In this study, we focused on UCN-01-mediated inhibition of proliferation in 3 human hepatoma cell lines: Huh7 is mutant for P53 and defective for P21, Hep3B is P53 defective, and HepG2 expresses wild-type P53/P21. We examined which pathways the three cell lines have in common, and we performed invasion studies using Huh7 cells and attempted to illustrate the mechanisms at the protein level.

## Methods

### Chemicals and antibodies

UCN-01 was purchased from Sigma-Aldrich, Inc. (St. Louis, MO USA). Phospho-anti-P53 and phospho-T68-anti-Chk2 polyclonal antibodies were purchased from Cell Signaling Technology (Beverly, CA USA). Anti-Cdc25c, CDK2, Chk2, Cyclin B1, p53, and beta-actin monoclonal antibodies were obtained from Santa Cruz Biotechnology (Santa Cruz, CA USA). The anti-p21^WAF1^ monoclonal antibody was obtained from Calbiochem (Cambridge, MA USA).

### Cell culture and UCN-01 treatments

The three different human HCC cell lines (HepG2, Hep3B, and Huh7) were grown at 37°C in the presence of 5% CO_2_ in Dulbecco's modified Eagle's media (DMEM) supplemented with 10% foetal bovine serum (FBS). Primary human hepatocytes were obtained from CellzDirect, Inc. (Tucson, AZ, USA) and cultivated in the manufacturer’s culture media. HCC cells and primary hepatocytes were treated with different concentrations of UCN-01 dissolved in dimethyl sulphoxide (DMSO). The cells were harvested, and whole cell extracts were used for western blots.

### Growth inhibition assay

Cell proliferation was analysed using the 3-(4, 5-dimethylthiazol-2-yl)-2, 5-diphenyltetrazolium bromide (MTT) assay. Briefly, the cells were seeded in a 96-well dish to a final concentration of 1×10 [4] cells/well and incubated in DMEM containing 10% FCS overnight. After incubation with different concentrations of the tested compounds for 72 h, the cells were incubated for 2 h with DMEM containing 0.4 mg/ml MTT. The conversion of MTT to formazan in metabolically viable cells was measured at 490 nm absorbance in a 96-well microtiter plate reader. The per cent conversion in mock-treated control cells was used to evaluate the effects that the chemicals had on cell growth and to determine the IC50 concentration.

### Cell cycle analysis

Exponentially growing cells were incubated with various concentrations of UCN-01 or treated with 0.1% DMSO as the vehicle control for 72 h. The cells were harvested, washed once with cold phosphate-buffered saline (PBS), fixed in ice-cold 80% ethanol, and stored at 4°C. Prior to analysis, the cells were washed again with PBS and suspended in 1 ml of a cold propidium iodide (PI) solution containing 10 μg/ml RNase A and 50 μg/ml PI. Next, flow cytometry was performed using the Beckman Coulter EPICS XL-MCL (Brea, US). The percentage of cells in various phases of the cell cycle was determined using FlowJo software (Ashland, US).

### Matrigel invasion assay

Hepatoma cell invasion was assayed in a 6-well Biocoat Matrigel invasion chamber (8 μm; BD Biosciences, Franklin Lakes, NJ USA). Two hundred thousand cells were resuspended in 1 ml of serum-free DMEM and were added to the upper surface of the invasion chambers. Hepatocyte growth factor (HGF; Richmond, VA USA) at 10 ng/ml was used as the chemoattractant and placed in the lower wells. After 72 h, the cells on the upper surface of the membrane were removed using cotton swabs, and the filters were fixed by immersion in 4% formaldehyde for 10 min. After two washes with water, the invaded cells were stained with haematoxylin-eosin (Fisher Scientific, PA USA). Excess dye was removed by rinsing twice with water. The cells that had passed through the Matrigel matrix and the 8-μm pores in the culture inserts were counted using light microscopy.

### Western blot analyses

The cells treated with various concentrations of UCN-01 were lysed with lysis buffer [20 mM Tris–HCl (pH 8.0), 150 mM NaCl, 2 mM EDTA, 100 mM NaF, 1% NP40, 1 μg /ml leupeptin, 1 l g/ml aprotinin, and 1 mM PMSF]. Cell lysate protein content was determined using a Bicinchoninic acid (BCA) protein assay kit (Pierce, Rockford, IL USA). The extracts (40 μg protein) were fractionated on polyacrylamide-SDS gels and transferred to PVDF membranes (Amersham, Arlington Heights, IL USA). The membranes were blocked with a solution containing 5% non-fat milk in PBS and incubated overnight with primary antibody at 4°C. Subsequently, the membranes were incubated with secondary antibody coupled to horseradish peroxidase (Amersham, Arlington Heights, IL USA). The reactive proteins were visualised using enhanced chemiluminescence (Amersham, Arlington Heights, IL USA) according to the manufacturer’s instructions. Western blot analyses were performed using standard procedures.

### Statistical analyses

All values are expressed as the mean ± SD. The significance between mean values was evaluated using the two-tailed unpaired Student's *t*-test (P < 0.05).

## Results

### Effects of UCN-01-mediated Growth Inhibition on Cell Lines

**Figure 1:**
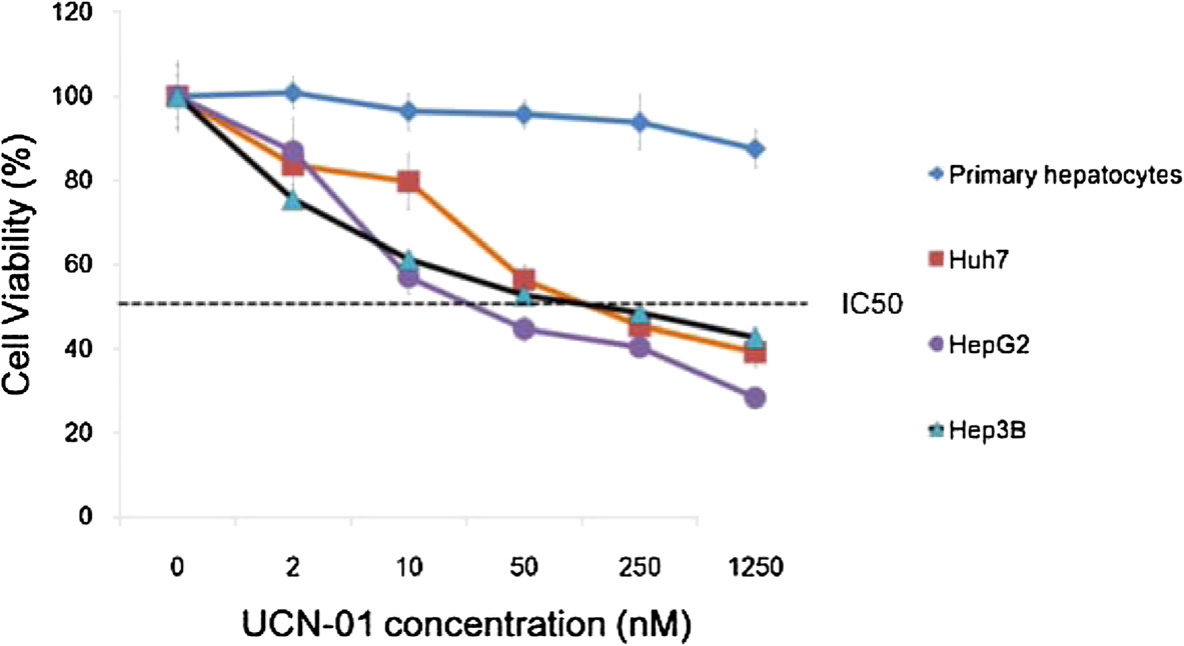
**UCN-01 inhibits growth of human hepatoma cells compared with human primary hepatocytes.** The cells were seeded (1×10 [4] cells/well) into 96-well plates and treated with different concentrations of UCN-01 or vehicle for 72 h. The value at each concentration represents the mean ± SD derived from triplicate tests.

The inhibitory effect on growth caused by UCN-01 in three hepatoma cell lines (HepG2, Huh7, and Hep3B) was evaluated using a cell viability assay (Figure 1). All cells were exposed to UCN-01 (0,2,10, 50, 250 and 1250 nM respectively) for 72 h and showed a dose-dependent growth inhibition. The experiment was repeated three times. Sensitivity levels to this drug, as assessed by the IC_50_ values, ranged from 69.76 to 222.74 nM (Table 1), and the HepG2 cell line was the most sensitive among the three cell lines tested.

**Table 1:** **IC**_**50**_**values of the growth inhibitory effects mediated by UCN-01 in the Huh7, HepG2, and Hep3B cell lines**

**Cell lines**	**IC**_**50**_**(nM)**
Huh7	222.74±13.35
HepG2	69.76±5.14
Hep3B	182.64±10.89

### Cell cycle analysis by flow cytometry

**Figure 2:**
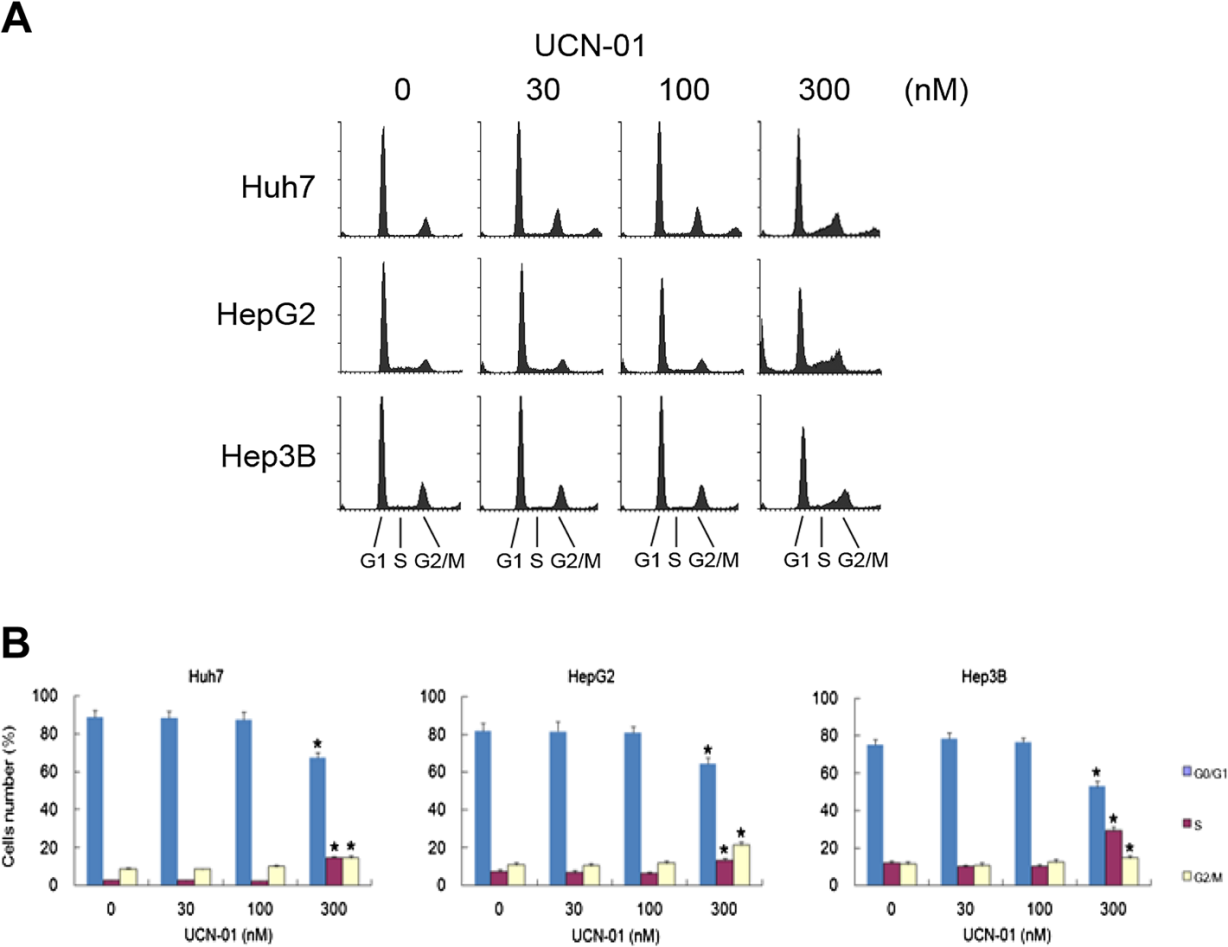
**UCN-01 treatment in hepatoma cells resulted in significant S phase and G2/M arrest.** Representative flow cytometric graphs are shown of cells treated with different concentrations of UCN-01 for 72 h. (**A**) Flow cytometry profiles of hepatoma cells are shown. Cell treatments were as follows: DMSO only and UCN-01 30 nM, 100 nM, and 300 nM. (**B**) The percentage of cells in G1, S, and G2/M phases illustrates that hepatoma cells accumulate in the S and G2/M phases after 300 nM UCN-01 treatment. The UCN-01-treated cells and untreated cells that differ significantly are indicated by and asterisk (P < 0.05).

The results of the cell cycle analyses are shown in Figure 2. UCN-01 induced arrest in the S and G2/M phases and increased the number of cells in the S and G2/M phases in all three cell lines. Huh7, HepG2, and Hep3B cells treated with UCN-01 for 72 h accumulated cells in the S and G2/M phases in a dose-dependent manner compared with untreated controls. Furthermore, the highest concentration of UCN-01 (300 nM) led to a significant reduction of cells in the G1 phase. PI-stained cells were assessed using flow cytometry with the FL-2 channel (Figure 2A). Treatment of the Huh7, HepG2, and Hep3B cells with 300 nM UCN-01 resulted in a significant increase in the percentage of cells in S phase (14.8%, 13.2%, and 29.3%, respectively) compared with the untreated cells (2.73%, 7.44%, and 11.1%, respectively; P < 0.05). The percentage of cells in the G2/M phase was also significantly increased in the Huh7, HepG2, and Hep3B cells after treatment with 300 nM UCN-01 (14.4%, 21.6%, and 14.7%, respectively) compared with the untreated cells (8.81%, 10.6%, and 11.4%, respectively; P < 0.05; Figure 2B). These results indicate that treatment with 300 nM UCN-01 induced arrest in S phase and G2/M phases in all three cell lines.

### UCN-01 induces cell cycle arrest in hepatoma cells via the p53/pP21^waf1^ and CHK2/CDC25C pathways

**Figure 3:**
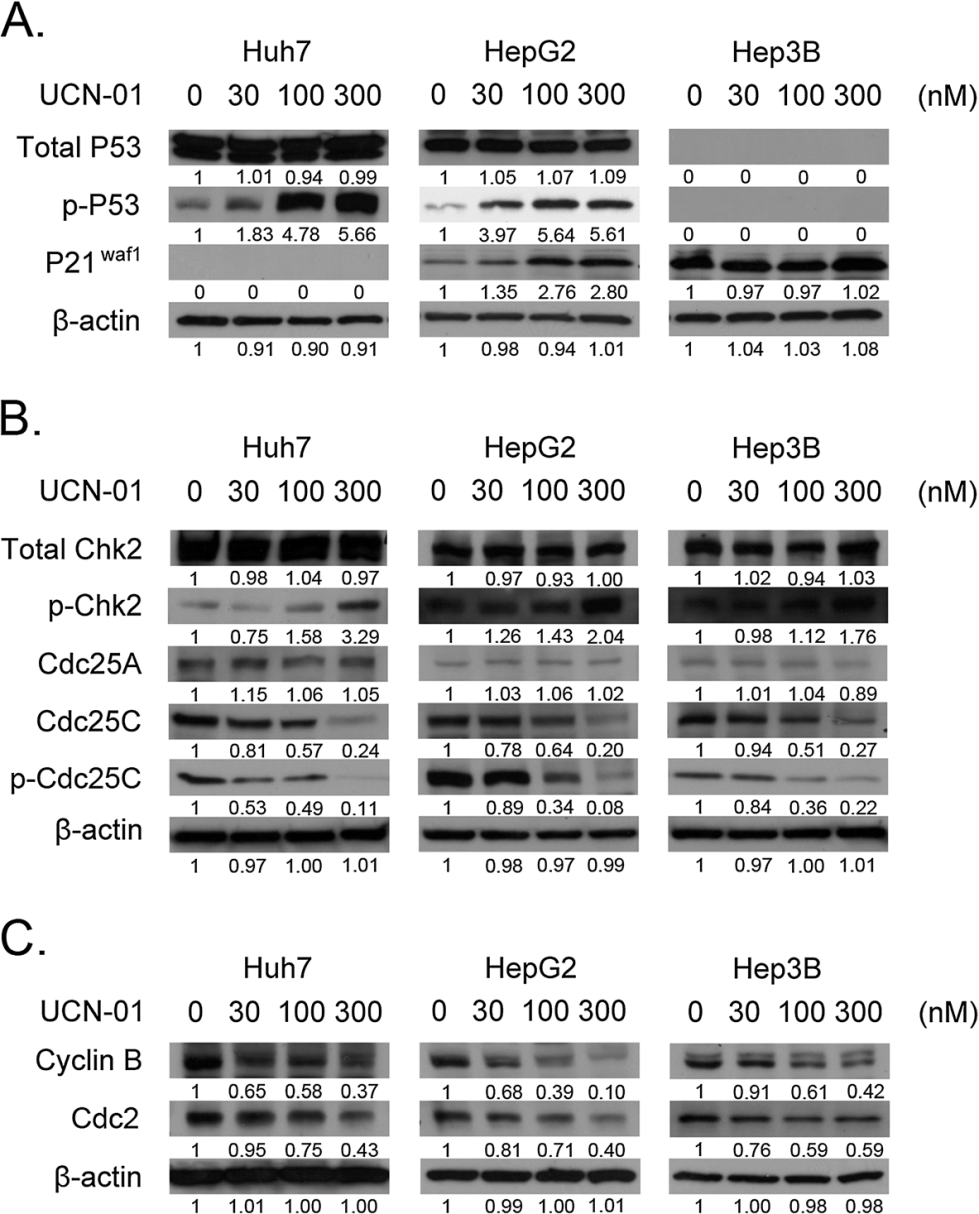
**Measurement of hepatoma cell protein levels after UCN-01 treatment.** Proteins were extracted and analysed through western blotting. β-actin was used as the loading control. (**A**) Western blots were performed for phospho-p53, p53, and p21^waf1^ in Huh7, HepG2, and Hep3B cells cultured with 0, 30, 100, and 300 nM UCN-01 for 24 h. (**B**) Total CHK2, phosphorylated CHK2, total cdc25a, and total cdc25c levels were detected. (**C**) Cyclin B was detected using a specific antibody. Cyclin B decreased in all three cell lines in a dose-dependent manner.

To understand the mechanisms by which UCN-01 induces G2/M phase arrest, we studied intracellular signalling through western blot analyses (Figure 3). UCN-01 has been reported to inhibit chk1 and abrogate the G2/M checkpoint in human colon carcinoma HCT116 cells and to induce G1 arrest in other human cancer cells; however, there are no reports on the effects of UCN-01 on hepatoma cell lines. Furthermore, we investigated UCN-01-induced G2/M phase arrest, which has not been studied previously. Thus, our work investigates the mechanism of UCN-01-induced G2/M phase arrest in Huh7, HepG2, and Hep3B cell lines. There are two key pathways involved in normal mammalian cell cycle arrest: p53/p21^waf1^ and CHK2/CDC25C. Figure 3A indicates that p21^Waf1^ expression increased in a UCN-01 dose-dependent manner in HepG2 cells but not in Huh7 or Hep3B cells. Increased p53 phosphorylation was observed in Huh7 and HepG2 cells, even at very low concentrations (30 nM) of UCN-01. However, total cellular p53 protein levels were not increased even up to 300 nM UCN-01 in any of the cell lines. Figure 3B indicates that CHK2 phosphorylation increased in a UCN-01 dose-dependent manner in all three cell lines, while CDC25C protein levels decreased in all cell lines. We also examined cyclin B levels in each cell line (Figure 3C). The Cdc2/cyclin B complex is the key enzyme regulating the G2 to M transition and is controlled by phosphorylation at various sites. Therefore, we monitored cyclin B levels in each cell line after treatment with UCN-01. The results show that cyclin B levels decreased in a dose-dependent manner in all three cell lines.

### Cell invasion is inhibited by UCN-01

**Figure 4:**
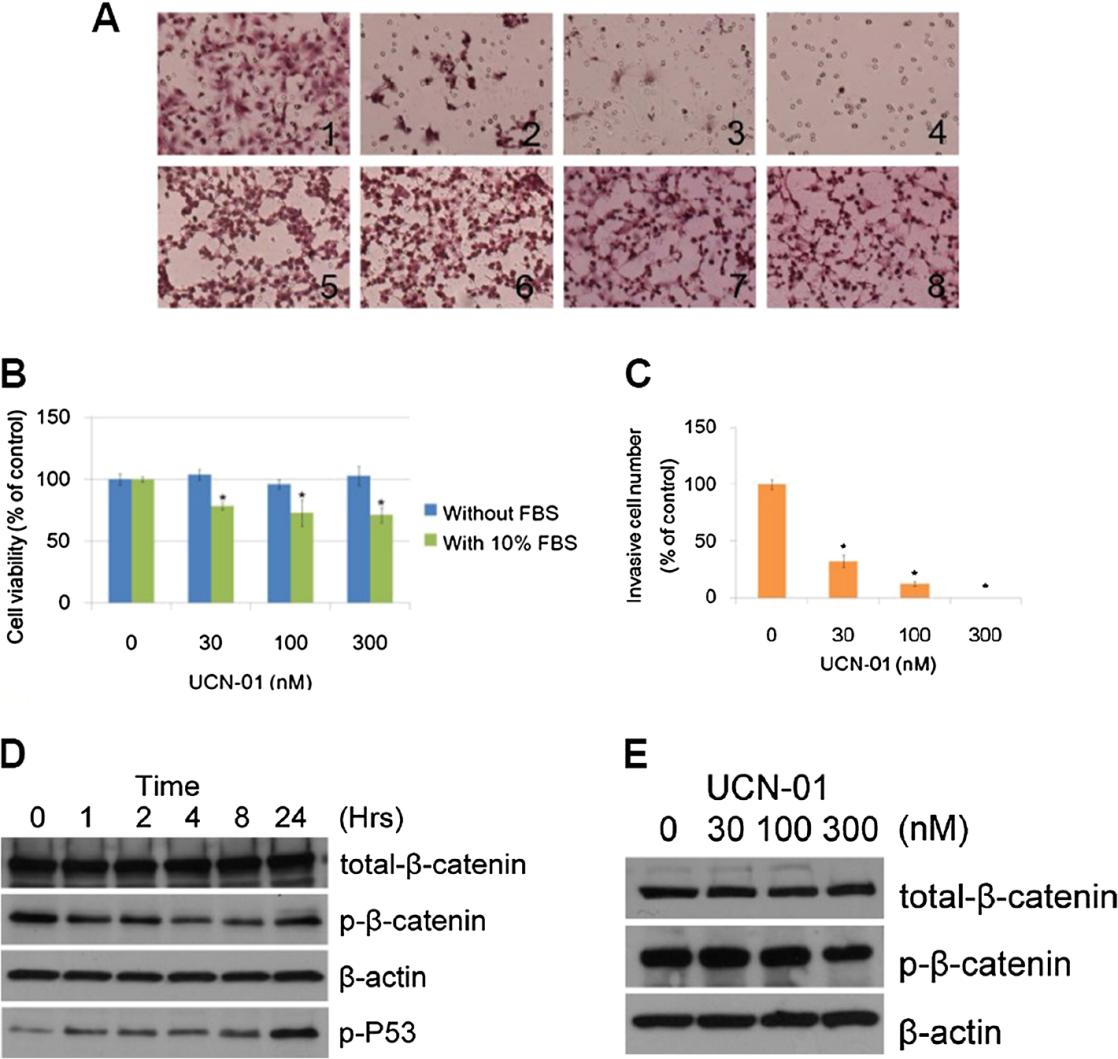
**UCN-01 reduces Huh7 cell invasion compared with untreated controls.** (**A**) A Transwell invasion assays were performed in the presence of HGF as the chemoattractant with the different hepatoma cell lines (Huh7, HepG2, and Hep3B) and different doses of UCN-01. After 72 h, the lower layer membrane was fixed and observed using the 400× objective. The areas (labelled with 1–8,) were observed using the 400× objective. The upper line shows that Huh7 cell numbers decreased in a UCN-01 dose-dependent manner (areas 1, 2, 3, and 4 correspond to 0, 30, 100, and 300 nM UCN-01, respectively). The lower line shows that there were no obvious changes in HepG2 (5 and 6 correspond to 0 and 300 nM UCN-01, respectively) and Hep3B (7 and 8 correspond to 0 and 300 nM UCN-01, respectively) cells. (**B**) The column graph represents the percentage of viable Huh7 cells cultured in serum-free media or 10% FBS after treatment with different UCN-01 concentrations. There were no significant differences between cells using the different concentrations of UCN-01 compared with untreated cells when cultured with serum-free media (P > 0.05). (**C**) The column graph represents the percentage of Huh7 cells that invaded through the transwell compared with the control. The values from each concentration represent the mean ± SD derived from triplicate tests. Significant differences between treated cells and the untreated cells are indicated by an asterisk (P < 0.05). (**D**) Total β-catenin, phosphorylated β-catenin, and phosphorylated p53 in Huh7 cells were detected using specific antibodies after treatment with 100 nM UCN-01. β-Actin was used as the loading control. Total β-catenin levels did not change after 24 h of UCN-01 treatment; however, phosphorylated β-catenin was down-regulated between 1 to 8 h of treatment, and the down-regulation was significant during 4–8 h of treatment. Phosphorylated p53 levels were gradually up-regulated from 1 to 24 hr of treatment.(**E**) Western blots of total β-catenin and phosphorylated β-catenin in the Huh7 cell lines. There was no significant change in total β-catenin or phosphorylated β-catenin after treatment with 0, 30, 100, and 300 nM UCN-01 for 24 h.

Hepatoma cell invasion was assayed using the Matrigel invasion chamber. After 72 h, the lower layer membranes were fixed and stained, and the number of cells invading through the membrane was counted (Figure 4A). Huh7 cells exposed to UCN-01 showed a significant decrease in the number of cells able to invade across Matrigel-coated membranes compared with untreated cells. In contrast, UCN-01 treatment of HepG2 cells and Hep3B cells did not affect their invasive ability. We compared the proliferation rates of Huh7 cells cultured in 10% FBS or in serum-free media. There were no significant differences between the total number of Huh7 cells in serum-free media treated with various UCN-01 concentrations (P > 0.05; Figure 4B). This result demonstrates that UCN-01-mediated invasion inhibition is not due to inhibition of proliferation. For the 30, 100, and 300 nM UCN-01-treated groups, the percentages of Huh7 cells invading through the membrane were 32.29%, 12.29%, and 0%, respectively. The percentage significantly decreased between UCN-01-treated cells and the control group (P < 0.05; Figure 4C). To understand how UCN-01 inhibits Huh7 cell invasion, we examined total β-catenin, phosphorylated β-catenin and p53, and active- β-catenin levels through western blot analyses. These results show that phosphorylated β-catenin is down-regulated by UCN-01 in a time-dependent manner from 0 to 8 h with a 100-nM UCN-01 treatment (Figure 4E). In contrast, phosphorylated p53 levels increased from 0 to 24 h with a 100-nM UCN-01 treatment. There was no change in total β-catenin and phosphorylated β-catenin levels after 24 h of the UCN-01 treatment (Figure 4D).

## Discussion and conclusion

In this study, we investigated UCN-01 antitumour activity in three different hepatoma cell lines with a particular emphasis on the mechanism of the G2/M cell cycle arrest induction and invasion inhibition in Huh7 cells. There were several novel findings presented here. (a) Growth of all three hepatoma cell lines was significantly inhibited by UCN-01 (each with a different sensitivity), whereas there was no effect on the normal hepatic cell line. (b) UCN-01-induced S and G2/M phase (and not G1) cell cycle arrest altered the p53 and CHK2 pathways. (c) Increased phosphorylation of Chk2 Thr68 and p53 was critical for UCN-01-induced G2 arrest, while total CHK2 and p53 remained the same. (d) In Huh7 cells, which are p53 mutant and p21 defective, and in Hep3B cells, which are p53 defective, S phase and G2/M cell cycle arrest is induced by UCN-01, suggesting that the G2/M arrest is p53 independent. (e) In UCN-01-treated Huh 7 cells, invasion activity was significantly inhibited, which may be correlated with the down-regulation of phosphorylated β-catenin at Ser 552.

UCN-01 was originally identified in *Streptomyces* as a selective protein kinase C inhibitor [19] and was subsequently found to inhibit many other kinases including cyclin-dependent kinase 2 (CDK2), Chk1, and, most recently, Akt [20-22]. By inhibiting Chk1, UCN-01 blocks the phosphorylation and proteosomal degradation of Cdc25c phosphatase [20,23,24]. Several phase I and II trials of UCN-01, either alone or in combination with established cytotoxic agents, are currently underway, and preliminary evidence of activity against certain malignancies has been reported [25-29].

There have been reports that UCN-01 inhibits the growth of various human cancers, e.g., leukaemia, colon, and pancreatic cancers, through the induction of a G1 arrest; [30-33] however, there are currently no reports on the effects UCN-01 has on HCC lines. In our study, UCN-01 effectively inhibited cell growth and viability in three human hepatoma cell lines in a dose-dependent manner. Cell cycle analysis revealed that UCN-01 inhibition of cell viability was caused by cell cycle arrest at the S and G2/M phases, accompanied by a decrease in the number of cells in G1. These results differ from the findings in other cancer studies [21,31,34]. In those studies, UCN-01-mediated induction of G1 arrest has been attributed to inhibition of CDK2, which results from CDK2 dephosphorylation at Thr160 and the induction of the CDK2-inhibiting proteins p21 and p27 in various cell lines [20,35,36].

Here, we focused on elucidating how UCN-01 induces G2/M arrest in Huh7, HepG2, and Hep3B cell lines. Among these cell lines, Huh7 is p53 mutant and p21 defective, and Hep3B is p53 defective, enabling us to determine whether the cell cycle arrest is p53 dependent.

The cyclin B/Cdk2 complex is critical in regulating the cell cycle transition from G2 to M phase and is controlled by phosphorylation at various sites [37]. In our study, significant decreases in cyclin B and CDC25, as well as an increase in phosphorylated CHK2, were observed after 24 h of UCN-01 treatment. Cdc25 is a positive regulator of the cyclin B complex, which is inhibited by Chk2-mediated phosphorylation [37]. Thus, UCN-01 may induce G2/M cell cycle arrest through the CHK2-CDC25-cyclin B pathway.

However, we found that UCN-01 induces p53 phosphorylation at Ser 15 and increased p21^waf1^ protein levels in the HepG2 cell line. As a crucial cell cycle regulator, the p53 tumour suppressor has an important role in the cellular response to platinum agents [38,39]. For example, 1,2-diaminocyclohexane-acetato-Pt (DACH) arrests p53 wild-type cells in G1 and mutant p53 cells in G2/M phase in ovarian cancer [40]. P53 transcriptionally activates a series of genes involved in both the G1-S and the G2/M transitions in response to genotoxic stress [41,42]. Among these genes, p21 is a well-established negative regulator of the G1/S transition. p21 also inhibits the CDK1/cyclin B complex and maintains the G2 arrest [43-45]. Our study shows that the p53/p21^waf1^ pathway is also involved in the G2/M cell cycle arrest of HepG2 cells.

Interestingly, when UCN-01 was used to treat Huh7 cells (p53 mutant and p21 deficient) and Hep3B cells (p53 deficient), the G2/M arrest is still observed. These findings suggest that, although these two pathways both contribute to cell cycle arrest, the CHK2-Cdc25c pathway may have a more important role in these p53-deficient cell lines.

**Figure 5:**
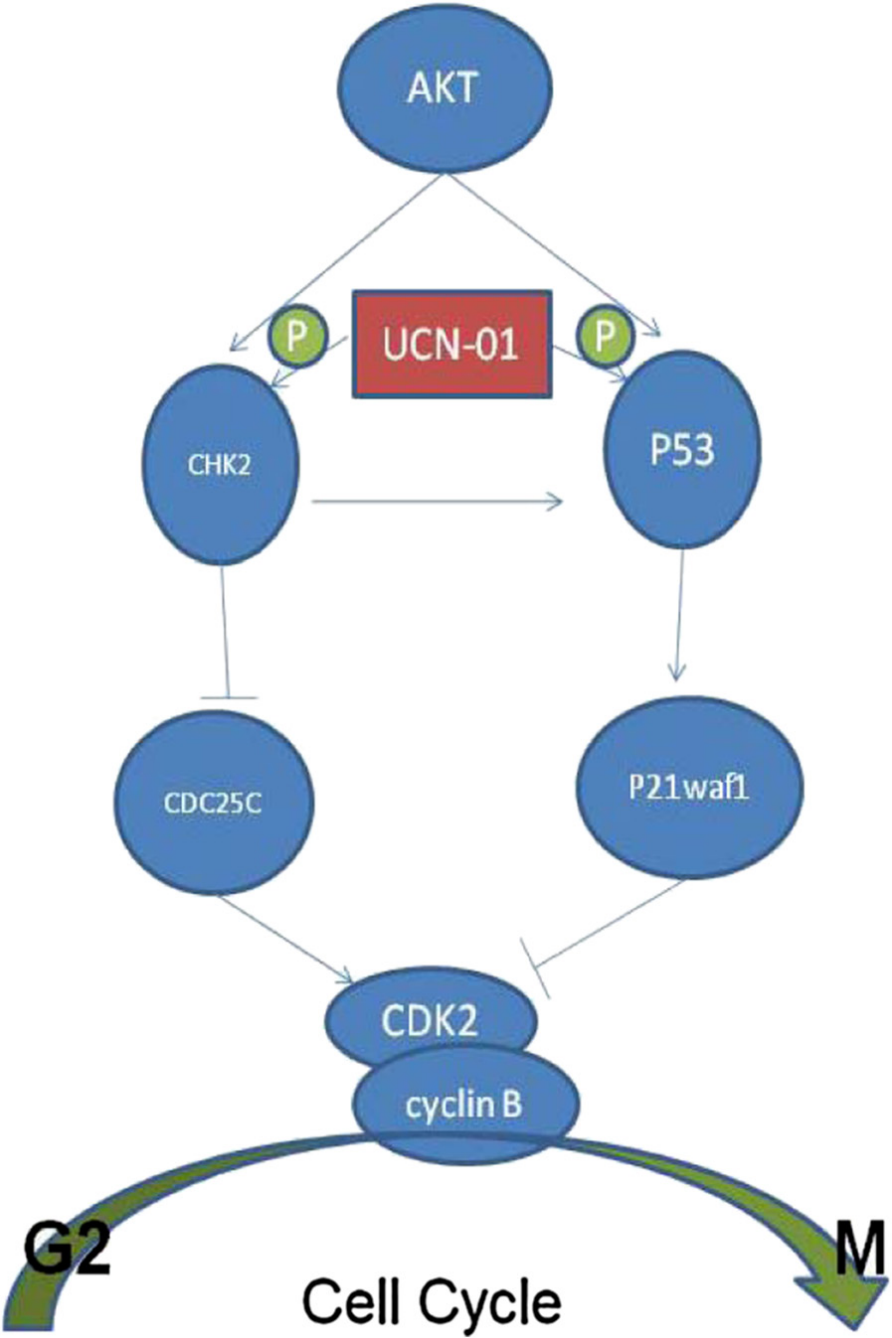
UCN-01 induces G2/M cell cycle arrest through the p53 and CHK2 pathways in hepatoma cell lines.

In our study, UCN-01 induces G2/M cell cycle arrest through these two pathways in the Huh7, HepG2, and Hep3B cell lines (Figure 5), but does not affect normal hepatocytes, which may be attributed to the slow turnover rate of normal hepatocytes.

Finally, we found that Huh7 cell invasive activity was significantly inhibited by UCN-01. Recent studies indicate that phosphorylated β-catenin has a critical role in the invasion and development of many different tumours [46-49]. β-catenin was originally identified as a component of cell-cell adhesion structures, interacting with the cytoplasmic domain of E-cadherin and linking E-cadherin to α-catenin. Phosphorylation of β-catenin causes its dissociation from these cell-cell contacts, and β-catenin accumulates in both the cytosol and nucleus, enhancing its interaction with 14-3-3ζ via a binding motif containing Ser 552 [46]. In our study, we examined total β-catenin and phosphorylated β-catenin levels at different time points, and found that phosphorylated β-catenin decreased 1–8 h after 100 nM UCN-01 treatment. Thus, inhibition of Huh7 cell invasion may be correlated with the down-regulation of phosphorylated β-catenin by UCN-01; however, further work is needed to confirm this finding.

Although recent studies indicate that UCN-01 binds to the human serum protein alpha1-acid glycoprotein and that this binding may hamper the ability of UCN-01 to inhibit cell proliferation and kill cells *in vivo*, UCN-01 has decent activity against several tumours at dose levels that are tolerated in clinical studies [27,28]. These findings indicate that UCN-01 is an effective agent for tumour cell proliferation inhibition *in vitro* and *in vivo* and may be clinically applicable for HCC treatment in spite of its binding to serum proteins.In summary, our results show that UCN-01 inhibited HCC cell growth through the induction of S and G2/M phase arrest. UCN-01-induced G2/M cell cycle arrest involved molecular alterations of cell cycle regulatory proteins in human hepatoma cells. Increased Chk2 phosphorylation at Thr68 was critical for UCN-01-induced G2 arrest. These findings indicate that UCN-01 modulates the G2 transition through the p53-p21^waf1^ pathway as well as the Chk2–Cdc25c–Cdc2/cyclin B1 pathway. We also found that cell invasion was significantly inhibited by UCN-01 in Huh7 cells, which may be related to the down-regulation of phosphorylated β-catenin. Overall, the current study demonstrates that UCN-01 may be a potential drug candidate for the treatment of hepatocellular carcinomas.

## Competing interests

The authors declare that they have no competing interest.

## Pre-publication history

The pre-publication history for this paper can be accessed here:

http://www.biomedcentral.com/1471-2407/13/167/prepub
