# Correction: UCN-01 induces S and G2/M cell cycle arrest through the p53/p21waf1 or CHK2/CDC25C pathways and can suppress invasion in human hepatoma cell lines

**DOI:** 10.1186/1471-2407-14-216

**Published:** 2014-04-04

**Authors:** Guoyi Wu, Linan Xu, Nan Lin, Bo Liu, Mark A Feitelson

**Affiliations:** 1 Department of General Surgery, The lingnan Hospital, The Third Affiliated, Hospital, Sun Yat-Sen University, GuangZhou 510630, PR China; 2 Department of Hepatobiliary Surgery, The Third Affiliated Hospital, Sun Yat-Sen University, GuangZhou 510630, PR China; 3 Department of Gynecology and Obstetrics, The First Affiliated Hospital, Sun Yat-Sen University, GuangZhou 510089, PR China; 4 Department of Biology, College of Science and Technology, Temple University, Philadelphia, PA 19122, USA

## Correction

After publication of this work [1], we noted that we inadvertently failed to include the complete list of all co-authors. In addition, it has been brought to our attention that an Authors Contributions section was not included in the published article. In this correction we rectify these mistakes. The full list of authors and the Authors Contributions section are reported below and the Acknowledgements section has been modified accordingly. We apologize for any inconvenience this oversight may have caused.

## Corrected Authors’ List

Guoyi Wu(1), Nan Lin(2), Linan Xu(3), Bo Liu(1) and Mark A. Feitelson(4)

1 Department of General Surgery, the lingnan Hospital, the Third Affiliated Hospital, Sun Yat-Sen University, GuangZhou 510630, PR China

2 Department of Hepatobiliary Surgery, the Third Affiliated Hospital, Sun Yat-Sen University, GuangZhou, 510630, PR China

3 Department of Gynecology and Obstetrics, the First Affiliated Hospital, Sun Yat-Sen University, GuangZhou 510089, PR China

4 Department of Biology, College of Science and Technology, Temple University, Philadelphia, PA 19122 USA

## Pre-publication history

The pre-publication history for this paper can be accessed here:

http://www.biomedcentral.com/1471-2407/14/216/prepub

## References

[B1] Guoyi W Linan X Nan L Bo L UCN-01 induces S and G2/M cell cycle arrest through the p53/p21waf1 or CHK2/CDC25C pathways and can suppress invasion in human hepatoma cell lines BMC Cancer 2013 13 167 10.1186/1471-2407-13-167 23537372 PMC3618254

